# Vaccination

**Published:** 1881-07

**Authors:** Henry M. Lyman

**Affiliations:** Professor of Physiology and of Diseases of the Nervous System, in Rush Medical College, Chicago, Ill.


CHICAGO MEDICAL

JOURNAL & EXAMINER.

VOL. XLIII.—JULY, 1881.—NO. 1.

### Original Communications.

ARTICLE I.

VACCINATION. By HENRY M. LYMAN, M.D., Professor of Physiology and of Diseases of the Nervous System, in Rush Medical College, Chicago, Ill.

It is very evident that much obscurity of thought regarding the origin and nature of vaccination still lingers in the minds of physicians. This is largely owing to the fact that a considerable period of time divides us from the epoch which was marked by the publication of Jenner's great discovery, so that many of the facts which were then widely known have now been nearly forgotten. But it is, perhaps, even more to be attributed to the circumstance that the principal authorities who have guided professional opinion were themselves ignorant of numerous facts which have been recently brought to light, and which serve to co-ordinate and to illuminate all previous observations. Hence it cannot be a matter of reproach that inadequate and erroneous opinions regarding this subject have been entertained by many fairly educated men, since even so eminent an author as Sir Thomas Watson, only a few months ago, found himself obliged, after reading Mr. Ceely's papers, which, though published forty years previously, had never before attracted his attention, to retract his life long utterances regarding the nature of bovine virus. The recent researches of Pasteur and of his companions, regarding bacterial infection, have also contributed greatly toward the solution of the mystery which, in the minds of many, seemed to invest the whole subject of protection against small-pox by vaccination. We are now, however, in a position to form a clear idea regarding a subject, in connection with which there is no longer any place for mystery.

The opinion was long entertained vaguely by many physicians that small-pox and vaccinia were entirely distinct, though similar, diseases. This belief was based upon the fact that Jenner did not himself fully comprehend the manner in which these two forms were related. He believed that they were identical in their origin, but he did not demonstrate that identity. It was his opinion that horse-pox infected the cow through the intervention of the hands of the stable-boy who attended both animals, but he did not establish the origin of the horse-pox. It was only when the experiment of variolating the cow with the virus of small-pox had been successfully performed that the fundamental identity of variola and vaccinia was demonstrated. This was first performed, in 1801, by Gasser, of Günsberg (Seaton, *Reynolds' System of Medicine*, Vol. I., p. 176, Am. Ed.) Inoculating eleven cows with small-pox virus, he produced on one of them vesicles bearing all the character of vaccine vesicles, and from them a stock of genuine vaccine lymph was obtained. The same experiment (*loc. cit.*) was performed, in 1802, at the Veterinary College at Berlin. Mr. Ceely, of Aylesbury, in England, was equally successful in 1839. In 1840 (*loc. cit.*) Mr. Badcock, of Brighton, succeeded in procuring genuine vaccine virus by variolation of the cow; and he met with equal success on thirty-seven subsequent occasions.

It has also been shown that cattle can be variolated by inoculation of the volatile exhalations of variolous clothing, etc., but this is of rare occurrence.

Another significant fact deserves mention in this connection. Since the general adoption of vaccination, the vaccine disease has almost entirely ceased to occur in the cow. Previous to the introduction of vaccination, when small-pox was one of the domestic pestilences, it was an exceedingly common thing to find cows suffering with the vaccine eruption upon the udder. It was never observed upon the bull. Nor was it discovered upon the cow, except during the period of lactation, when subjected to handling. This indicates that variola is not a primary bovine disease, but is communicated from the human sufferer to the lower animals. The identity of variola and of vaccinia (so far as their origin is concerned), and the dependence of cow-pox upon previous human small-pox may therefore be considered a demonstrated fact. Cases like that of the Beaugency heifer may be most probably explained by supposing that they originated in an infection communicated by the hand of a variolous tramp, or of some other milker who was the unsuspected bearer of the virus of small-pox.

The above mentioned experiments and observations also place in bold relief the fact that the manifestations of variola may be exceedingly varied by the conditions of their occurrence. The phenomena of inoculation for small-pox had taught this lesson long before the discovery of Jenner; and the varieties of post-vaccinal small-pox have illustrated the same thing ever since. Few diseases are more easily varied by external conditions. Thus we have discrete, confluent, and hæmorrhagic small-pox, post-vaccinal small-pox or varioloid, bovine small-pox, and vaccine disease—all springing from one common source. It is true that the identity of variolæ vacciniæ and human small-pox has been denied by Fleming and by others; but I think no unprejudiced person can read Ceely's original papers without coming to the same conclusion that was realized by Sir Thomas Watson, when he for the first time perused them, a few months ago—that they are actually one and the same disease, only modified by the different environments in which they occur. This experimental proof also corresponds with the deductions derived from observation of the protective effect of vaccination. There is no other example of immunity against any particular disease acquired by experience of some other disease. Scarlet fever protects against scarlet fever, measles against measles, typhus fever against typhus fever, small-pox against small-pox. If, then, vaccine disease protects against small-pox, every analogy goes to show that it protects because it is, indeed, itself, only a form of small-pox. As long as we allowed ourselves to consider it a separate disease, it was impossible to refute the argument of the homœopathist, who quoted vaccination as an example of a disease being remedial against another *similar* disease. Like cures, or rather, prevents, like in this case; therefore the law *similia similibus curantur* must be true. Such was the argument, and it could not be successfully attacked by those who held to the dual nature of these diseases. But when we accept the evidence of experiment in the variolation of cows, we can reply to the homœopathist: This is no case of like and like. Small-pox protects only against itself. Variolæ vacciniæ and variola are radically and essentially the same disease, and the one must necessarily protect against the other by reason of their actual identity.

It was not, however, until the recent experiments for the protection of animals and fowls against anthrax and chicken cholera, that all difficulty was removed from consideration of the problem. Pasteur, Toussaint, and others, in France and Germany, found that by *attenuating* the virus of chicken cholera, and by heating the blood or other liquids of anthrax, a dilute form of the specific poison was produced, which, when injected into a healthy animal, excited a modified, and generally non-fatal form of the disease, by which the animal was completely protected against the fully virulent form of the contagion. Here, then, we find an explanation of the manner in which the virus of bovine variola differs from the virus of human variola—it is variolous virus attenuated, diluted or modified by the action of the bovine tissues, so that, while it does not excite the general eruption of variola in the human subject, it does yet, when used for vaccination, so sufficiently modify the human tissues that they become tolerant of the fully virulent poison of unmodified human small-pox.

It follows, therefore, that we have in the variolation of the cow an ever-present method of originating fresh supplies of vaccine virus—a method which in time of need is much more reliable than the search for accidental cases of sporadic cow-pox, both because of the danger of confounding cow-pox with vaccinella, a puerperal eruption of the cow which has no relation whatever with small-pox, and because of the great rarity of genuine sporadic cow-pox in these days of limited prevalence of human small-pox. The discredit which some have sought to throw upon this method of produring vaccine virus grows out of ignorance of the fact that the virus yielded by the pustule of a freshly variolated cow is exceedingly energetic. It is so active that Watson and others long supposed that it was merely the original variolous matter transplanted back again from the cow to the human subject. Ceely found that it produced such acute symptoms that it was necessary to attenuate it by several successive human vaccinations before it was sufficiently moderate in its effects to be used with comfort. Such virus sometimes produces an actual eruption, closely simulating varioloid; but after a few successive vaccinations it becomes so far "humanized"—that is, *attenuated*—that it only occasionally causes such general disturbance. Sometimes, however, as I have this past winter observed, after the use of bovine virus, we get an eruption of numerous vesicles around the point of vaccination. In one instance I observed an attempt at general eruption, which, had it occurred after the use of virus from a freshly variolated cow, might have been mistaken by an ignorant person for an aborted variolous exanthem. Similar eruptions after the use of bovine virus are mentioned by all the writers on vaccination; and they would no doubt be more frequent if the original bovine virus (Beaugency, or other stock) had not been already diluted by numerous successive calf-inoculations before it reached the profession.

The superior value of bovine virus for purposes of vaccination is now fully established by the results of accurate observation in England and Europe. It was foreseen by thoughtful men very soon after the announcement of Jenner's discovery, that the protective influences of vaccination must necessarily diminish with the progress of time; but the enthusiastic advocates of vaccination would not listen to any such opinions. For a number of years, in fact, the protection against small-pox seemed to be almost perfect. But a quarter of a century had not elapsed before it was remarked that post-vaccinal small-pox was becoming frequent. It was ascertained, at length, that by multiplication of the points of vaccination a greater degree of immunity was conferred. For a long time insufficiency of vaccination was claimed as the cause of post-vaccinal small-pox. But as years passed on, it was found that multiplication of marks, though better than single puncture, was becoming diminishingly protective. At the same time, the mortality of small-pox in unvaccinated persons was also gradually increasing. The following table is given by Dr. Cameron (*Fortnightly Review*, May, 1881):

| Percentage of mortality in small-pox in persons showing | 1836–51 | 1852–67 | 1870–79 |
|---|---|---|---|
| Cicatrices of vaccination | 6.9 | 7.6 | 9.2 |
| " 1 | 9.2 | 14.8 | 13.65 |
| " 2 | 6.0 | 8.7 | 10.14 |
| " 3 | 3.6 | 3.7 | 7.4 |
| " 4 or more | 1.1 | 2.0 | 4.83 |
| " 1 or 2 | 7.9 | 11.5 | 10.29 |
| " 3 or more | 2.4 | 2.8 | 5.8 |
| Percentage of mortality in small-pox, or in unvaccinated persons. | 37.5 | 35.7 | 44.6 |

During the earlier years of the century the mortality from post-vaccinal small-pox did not exceed one per cent. It has gradually increased, with temporary abatements, caused by improved hospital accommodation, treatment, etc., but on the whole, steadily increasing.

The causes of this result are two-fold: One great cause lies in the gradual loss of the protective influence of hereditary tolerance which had been acquired by an indefinitely long period of exposure to small-pox. This subject I have elsewhere discussed (*The Medical Record*, June 26, 1880, p. 720). The other causes which we at present have to consider, consists in the gradual modification of vaccine virus itself by repeated transmission to healthy subjects. In England, whence the above statistics are derived, vaccination is performed with human lymph, the greater part of which has descended directly from the original bovine virus introduced by Jenner. In other words we have here the results of a series of "culture experiments," in which bovine virus has been for many generations cultivated in the liquids of successive healthy human bodies. We find in these cultivations a regular progress toward a result analogous to the result of successive cultivations of the *bacillus anthracis* in healthy fluids outside of the animal. Gradually the bacillus loses its infective energy, until at length it becomes utterly innocuous, like the harmless hay-bacillus from which, by appropriate management, it was originally transformed. This process is evidently going on in the case of English vaccine virus. At its present rate of progression it is not difficult to estimate approximately the period necessary to reduce such virus to a condition of non-protective inertia. The bovine virus which we now use in this country has not yet had time to degenerate appreciably; but there is no reason to suppose that its course, whether cultivated in the body of the calf, or in the body of the human infant, will vary from the course of Jenner's original bovine virus in England.

The only sufficient remedy for this deterioration consists in occasional reversions to the original product of the variolated cow. By a few subsequent calf cultivations, this can be sufficiently attenuated for human use; and thus the original protective power of vaccination, so far as energy of the virus is concerned, may be maintained. Of course this cannot in any way compensate for that loss of hereditary tolerance of small-pox which is steadily progressing.

One more lesson we may learn from these observations. It is evident that small-pox is a disease which does not prevail among us as a direct transmission from some indefinitely remote first case of variola. It must necessarily be continually originating, *de novo*, in some part of the world. Were this not the case, small-pox, by reason of constantly progressive dilution, attenuation, or modification, of its virus by successive cultivation in the bodies of the healthy, would long since have lost its virulent character, and would have ceased to exist. We are justified in the adoption of this conclusion by what may be observed in any protracted epidemic of the disease. As Marson remarked, long ago, such epidemics cease not so much by reason of a lack of unaffected individuals in a community, as by reason of causes which he was not then in a position to explain. A sufficient explanation of the fact may be found in the diminishing energy of variolous virus by reason of frequent transmission through the bodies of healthy individuals. Just as the virulent energy of the *bacillus anthracis* diminishes, and finally disappears, as a result of its cultivation in successive portions of a healthy cultivating fluid, so the variolous virus finally loses so much of its virulence during the course of an epidemic that, though capable of producing the typical phenomena of small-pox as long as it produces any effect, the newly formed virus becomes less and less capable of maintaining its specific existence, and *dies out*, as we say.

It is true that we might not be at liberty to draw this conclusion from observation of variolous epidemics alone; but such observations, taken in connection with what we now know regarding the fate of a certain definite body of variolous virus—the modified Jennerian bovine virus, studied in England during the present century—enable us to arrive at certain definite scientific conclusions of the highest importance.

I am well aware that the revivification of variolous virus which takes place whenever it is transported to a virgin soil in a susceptible population, seems to contradict the hypothesis of its deterioration by transmission from person to person. This seeming contradiction, however, is more apparent than real. If, by mere transplantation, a virus could be restored to its original degree of energy, every virulent disease should long since have become everywhere endemic within its own climatic zone; and it should now be impossible to trace the march of any epidemic. Instead of this, we can trace the course of small-pox on lines which always radiate from an oriental, endemic focus where there is every reason to suppose that the disease is continually arising, *de novo*, as a result of special local reactions between man and his environment.

